# Histopathology diagnosis of coeliac disease – clinicopathological correlation is key!

**Published:** 2015

**Authors:** Marjorie M Walker

**Affiliations:** *University of Newcastle, Faculty of Health and Medicine, School of Medicine & Public Health Callaghan NSW 2308, Australia*

**To The Editor:**

A robust diagnosis of coeliac disease is paramount for patient and clinician. The recent paper and subsequent editorial in this journal emphasises this point (1,2). However there is little mention of recent evidence based international guidelines in considering which classifications to use (3-5). These guidelines are carefully considered by the authors, have undergone rigorous review and are backed by the literature and outline both definitions of coeliac disease (3) and guidelines for diagnosis and management (4, 5). 

Why is it so important to make a cast iron diagnosis in coeliac disease? For the patient, treatment with a gluten free diet is costly, socially restricting and difficult, as many foods may have hidden gluten. For the clinician, the responsibility lies in prevention of future complications, ranging from the consequences of malabsorption such as osteoporosis to a possible malignant outcome. It is important that histopathologists, *in tandem* with clinicians, make an early diagnosis. For histopathology the difficulties often lie in making this diagnosis at an early stage. Two studies underline the importance of suggesting coeliac disease when the histopathological features do not include flat mucosa (6, 7). Patients with mild enteropathy may experience clear gluten-induced symptoms and benefit from a gluten free diet (6) and also have weight loss, anaemia, folate deficiency, hyperparathyroidism and evidence of osteoporosis (7). Aside from performing serology (and where needed HLA testing), endoscopists should take enough biopsies to allow us to make the diagnosis – at least 4-6, including duodenal bulb – to make a diagnosis as this increases the chance to spot early and histological features(8).

We should also be mindful that normal architecture and ≥25 IELs/ 100 enterocytes (lymphocytic duodenosis (4) can represent coeliac disease in some patients, given the right clinico-pathological setting. Histopathologists should suggest this diagnosis when there is no current serology / HLA typing available to prompt these inveatigations. Although many other conditions are associated with LD (4,9), we must not ignore this finding. Mild enteropathy is not mild disease!
